# Anodal HD-tDCS on the dominant anterior temporal lobe and dorsolateral prefrontal cortex: clinical results in patients with mild cognitive impairment

**DOI:** 10.1186/s13195-023-01370-y

**Published:** 2024-02-03

**Authors:** Soheila Rezakhani, Mahmood Amiri, Atefe Hassani, Khadijeh Esmaeilpour, Vahid Sheibani

**Affiliations:** 1 Neuroscience Research Center, Institute of Neuropharmacology, Kerman University of Medical Sciences, Kerman, Iran; 2 Medical Technology Research Center, Institute of Health Technology, Kermanshah University of Medical Sciences, Kermanshah, Iran

**Keywords:** Mild cognitive impairment, HD-tDCS, Left dorsolateral prefrontal cortex, Dominant anterior temporal lobe, MoCA

## Abstract

**Objectives:**

Mild cognitive impairment (MCI) is a neurocognitive disorder in which the cognitive and mental abilities of humans are declined. Transcranial direct-current stimulation (tDCS) is an emerging noninvasive brain stimulation technique aimed at neuromodulation. In this study, we investigate whether high-definition anodal tDCS stimulation (anodal HD-tDCS) in MCI patients in two different brain regions will be effective in improving cognitive function.

**Methods:**

This study was done as a randomized, double-blind clinical trial. Sixty MCI patients (clinically diagnosed by expert neurologists) were randomly divided into three groups. Two groups received 2-mA anodal HD-tDCS for 20 min for 2 weeks (5 consecutive days in each week, 10 days in total). In the first group (twenty patients), the left dorsolateral prefrontal cortex (left DLPFC) was targeted. In the second group (twenty patients), the target zone was the dominant anterior temporal lobe (DATL). The third group (twenty patients) formed the Sham group. The Montreal Cognitive Assessment (MoCA) and Quality of Life in Alzheimer’s Disease (QoLAD) were considered as the outcome measures.

**Results:**

MCI patients obtained the highest MoCA mean scores in both left DLPFC and DATL groups versus the study baseline 2 weeks after the intervention. In addition, the MoCA mean scores of MCI patients were greater in both intervention groups compared to the Sham group up to 3 months post-stimulation (*p*-value ≤ 0.05). However, as we moved away from the first stimulation day, a decreasing trend in the MoCA mean scores was observed. Moreover, in the left DLPFC and DATL groups, higher QoLAD mean scores were observed 3-month post-stimulation, highlighting the effectiveness of anodal HD-tDCS in improving the quality of life in MCI patients.

**Conclusion:**

In this research, it was shown that applying anodal HD-tDCS at left DLPFC and DATL brain regains for two successive weeks improves cognitive function in MCI patients (by obtaining higher values of MoCA scores) up to 3 months after the intervention compared to the Sham group. This illustrates the positive effects of HD-tDCS, as a non-pharmacological intervention, for improving cognitive function and quality of life in MCI patients.

**Significance:**

Two weeks after anodal HD-tDCS of the DLPFC and DATL brain regions, the MCI patients achieved the highest MoCA mean scores compared to the Sham group across all measurement intervals.

**Supplementary Information:**

The online version contains supplementary material available at 10.1186/s13195-023-01370-y.

## Background

People’s mental capabilities are impaired mostly due to neurological disorders and injuries, which can be treated via rehabilitation to some extent. Alzheimer’s disease (AD) is a widespread age-related neurological illness, particularly in the eighth decade in life and beyond, which is primarily characterized by mild memory loss [1, 2], apathy, depression, anxiety, and irritability [2–5]. According to a report from the Alzheimer’s Association, 70% of dementia cases are caused by AD worldwide [6].

Mild cognitive impairment (MCI) refers to a condition in which cognitive functions are slightly reduced compared to the previous conditions and appears to be a transitional period between normal aging and the clinical diagnosis of AD [3, 7–9]. Indeed, this cognitive impairment has adverse effects on quality of life and functional ability. MCI patients may experience memory losses such as loss of judgment-related memory, but these do not significantly interfere with their daily life activities [10, 11]. In this way, early detection and intervention at the MCI phase can delay the onset of dementia [2]. However, there is no conclusive indication that drug interventions can prevent cognitive decline and dementia [12].

AD and MCI share a complex and intricate relationship that has been extensively studied in the field of neurodegenerative diseases [13]. Typically, AD progresses through a preclinical phase with underlying biomarker abnormalities, then a prodromal state of MCI, and finally AD dementia. Annually, 10–15% of patients diagnosed with MCI give rise to AD dementia [13]. Identification of factors contributing to progression from MCI to AD is crucial for clinical prognostication and risk stratification to guide counselling and selection of potential treatments.

Brain electrical stimulation has long been of interest to medical and scientific groups. Transcranial direct current stimulation (tDCS) has appeared as a helpful method for noninvasively modulating brain activities in recent years [14]. Indeed, tDCS is a method that uses a low-intensity electrical constant current through electrodes placed on the scalp. This electrical current leads to changes in the extracellular milieu which, in turn, modulates the resting membrane potential of the neuronal populations in the stimulated cortical regions [15]. Short-term effects of tDCS occur through non-synaptic mechanisms by altering neuronal membrane potentials, while long-term effects likely occur through NMDA-dependent mechanisms and appear to be consistent with synaptic plasticity [16]. The anodic or cathodic stimulation causes excitatory or inhibitory facilitation effects of the cortical activities in the specified area, respectively. In anodic stimulation, membrane depolarization occurs, which enhances the continuity of transmission of the electrical impulse. In the case of cathodic stimulation, there is a hyperpolarization of the membrane, which allows greater inhibition of cell activities. It should be pointed out that the tDCS does not generate the action potential due to its low intensity, but it can facilitate the conductivity of the ion channels so that neuromodulation/plasticity may occur. The great advantages of tDCS include noninvasive, painless, easy-to-use, portable, and low-cost rehabilitation technique with minor side effects [5, 17, 18]. Current applications of tDCS in specific areas of the cortex may enhance memory and learning, and these effects not only occur during the stimulation period but also usually continue for several hours or days after the stimulus session [19].

As smaller electrodes increase the accuracy of current delivered to the targeted brain region, high-definition transcranial direct current stimulation (HD-tDCS) is preferred to traditional tDCS. In this way, neuromodulatory effects outside the target area are minimized [20, 21]. However, the HD-tDCS device is more expensive in comparison to the conventional tDCS. The smaller surfaces lead to increased current density. Therefore, for safety reasons, less current is applied. The safety of HD-tDCS and its efficacy in improving motor function and working memory have been reported in several studies [22, 23]. Until recently, most studies have made use of traditional tDCS (one anode and one cathode), and a few studies used HD-tDCS but have shown promising results [24, 25].

The prefrontal cortex is the main brain structure related to executive functions and includes the dorsolateral prefrontal cortex (DLPFC), medial, and orbitofrontal/VMPFC regions [26]. Although it is highly interconnected, it has been suggested that the DLPFC is more specialized in working memory [27]. Previous studies have also shown that tDCS may cause certain changes in neurophysiological and psychophysiological activity in the brain’s target areas [28, 29]. In this way, the results of a systematic review provided evidence of improved memory and executive function through non-pharmacological interventions in elderly people with MCI [30]. *Hakuei *et al*.* studied the influence of tDCS on executive function over the right inferior frontal gyrus and pre-supplementary motor area in young and older healthy adults. The results showed that tDCS can improve executive function especially decision-making and inhibitory control for older people [31]. Furthermore, studies of patients with mild vascular dementia, using the anodal tDCS method over four consecutive sessions (for 20 min), showed positive effects on short-term visual memory, verbal memory, and executive control [32]. Stimulation to the DLPFC has been applied by some studies, and enhancing working memory in healthy adults was observed [33, 34]. The results of two other studies on MCI patients revealed that tDCS may increase memory function in these patients [35, 36], but its effectiveness was not investigated for a long intervention.

In this study, 60 MCI patients were randomly divided into three groups. Two groups received 2-mA anodal HD-tDCS for 20 min for 2 weeks (5 consecutive days in each week, 10 days in total). In the first group (twenty patients), the left DLPFC was targeted. In the second group (twenty patients), the dominant anterior temporal lobe (DATL) was stimulated. The third group (twenty patients) formed the Sham group. The Montreal Cognitive Assessment (MoCA) and Quality of Life in Alzheimer’s Disease (QoLAD) were utilized as the outcome measures. We have made the following contributions:

- First, comparing the effects of anodal HD-tDCS in two different areas that contribute to memory functioning (left DLPFC and DATL), with Sham stimulation which may be helpful to detect the best stimulation target in MCI patients
- Second, increasing the intervention interval to 10 sessions, 5 consecutive days per week, and following up the patients for 3 months

## Materials and methods

### Study design

The experimental protocol was approved by the Ethics Committee of Kermanshah University of Medical Sciences in Iran. This study was a prospective, randomized, double-blind clinical trial with registration number IRCT 20130812014333N163. Before the experiment, all the subjects received a complete description of the experimental procedure and then signed written informed approval.

### Subject and setting

This study was carried out at the Neurosciences Research Center, Kerman, Iran. An expert neurologist who has successfully completed the official MoCA training provided a diagnosis of MCI. For consistency, the average age, the number of years of education, and medical history were comparable between the groups. Sixty right-handed participants had to (a) be aged between 55 and 90 years, (b) achieve a score of 4 or below on the BDI (Beck Depression Inventory) test, (c) obtain a score between 17–25 on the Montreal Cognitive Assessment (MoCA), and (d) pass the criteria of Peterson’s test [37]. Additionally, magnetic resonance imaging (MRI) was conducted to ensure that patients could not have comorbidities other than MCI. To be eligible for inclusion, patients had 4 or more years of primary education with a 1-year duration of MCI symptoms and be able to attend 10 stimulation sessions on 5 consecutive days. Participants with epilepsy, any medication affecting the central nervous system, history of neurological disease other than MCI, neurological focal defects, skin diseases (e.g., eczema, skin lesions), intracranial surgery, intraoral (extracorporeal) metal such as cracks, surgical clips, any implants such as cardiac pacemakers, vagus nerve stimulation (VNS), cochlear implants, and recurrent or severe headaches were excluded.

### Sample size and sampling method

**Fig. 1:**
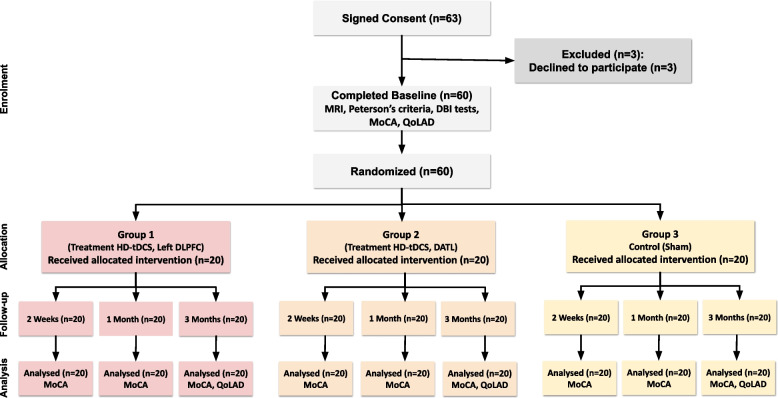
Schematic illustration of the experimental procedure. Sixty participants signed consent. There were three participants excluded before completing the baseline. Sixty participants were randomized and then divided into three groups. 2-mA anodal HD-tDCS for 20 min was applied for 2 weeks (5 consecutive days in each week) on the left DLPFC (20 patients, first group) and on the DATL (20 patients, second group). The third group (20 patients) received the Sham stimulation. All 60 patients completed a 2-week treatment and 1-month and 3-month follow-up. The CONSORT/2010 statement was utilized as a model to construct this figure

Following the previous study [38] and considering a 95% confidence interval, a power level of 80% with an effect size of 65%, the sample size for each group, was determined to be 20 subjects per group using G*Power software [39]. Figure 1 illustrates the outline of the study. Randomization was performed using computer random numbers. After completing initial assessments, participants were randomly assigned to the first intervention group with electrode placement on the left dorsolateral prefrontal cortex, the second intervention group with electrode placement on the dominant anterior temporal lobe, and the Sham group as the third group. The patients and the researchers (and the evaluating physician) were blinded; however, the technician applying the tDCS therapy and the person who performed the statistical analysis were not blinded. The participants were not permitted to interact with each other during their visits in any of the study phases. The CONSORT (Consolidated Standards of Reporting Trials) statement was employed to enhance the reporting of randomized controlled trials [40]. The MoCA and QoLAD were considered as the main outcomes, which were measured by following up the patients for 3 months.

### Procedure

**Fig. 2:**
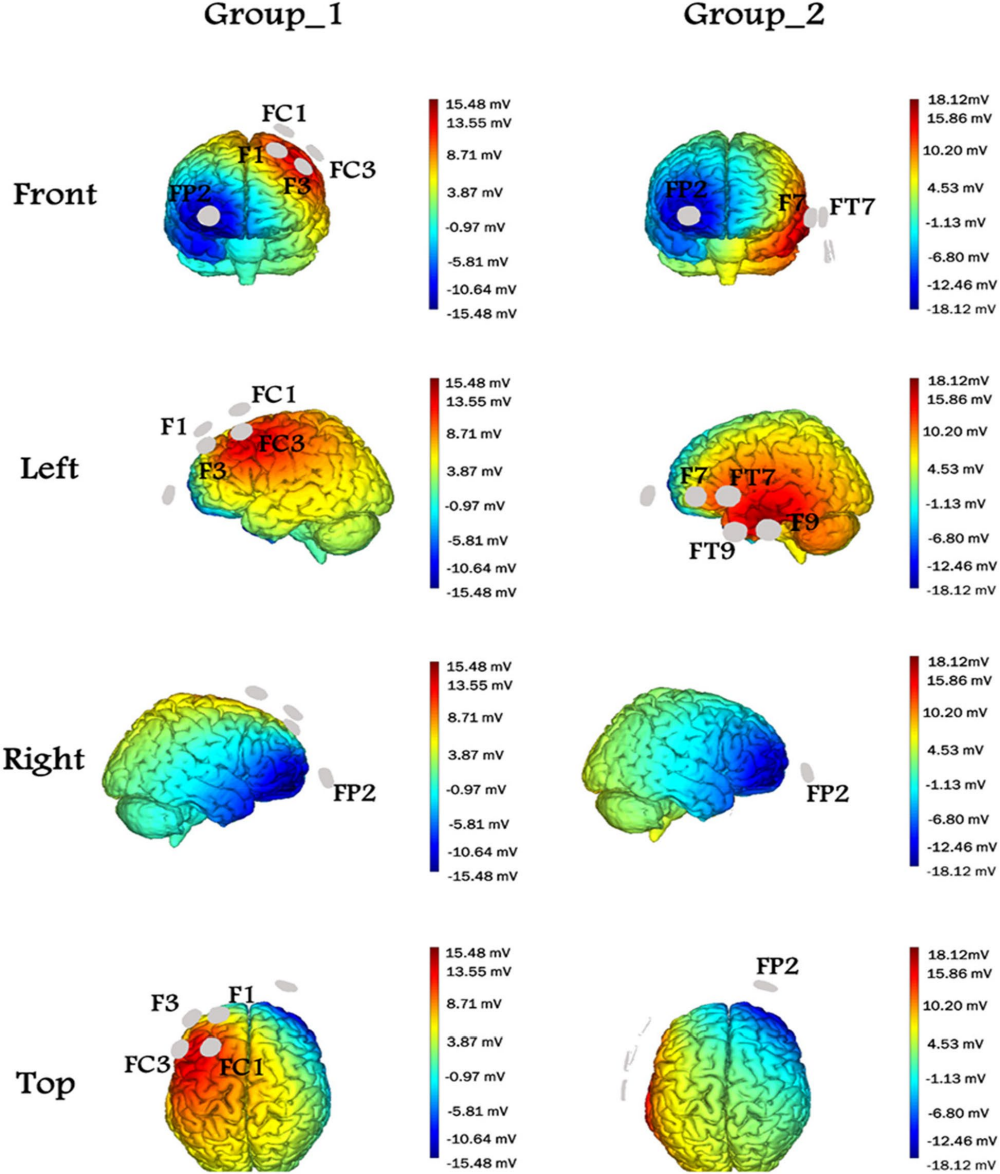
Two different 4 × 1 anodal HD-tDCS interventions. The position of electrodes is based on the international 10–10 system. Indicated locations were used for stimulation (blue: cathode; red: anode). For “Group_1,” the target zone was located on the left dorsolateral prefrontal cortex, and the electrode montage was as follows: cathode placed on the Fp2 location and the other four anode electrodes placed at F1, F3, FC1, and FC3 locations. For “Group_2,” the target zone was in the dominant anterior temporal lobe, and the electrode montage was as follows: cathode positioned on the FP2 and the other four anode electrodes on F7, FT7, T9, and FT9. The Neuroelectrics Instrument Controller (NIC2) software was used to create this figure

All the participants were subjected to neurological examination and blood tests to remove secondary causes of dementia or cognitive deficits such as hypothyroidism, liver or kidney disease, AIDS, vitamin B12 deficiency, folate, and syphilis. Brain magnetic resonance imaging (MRI) was performed before intervention to rule out focal or lacunar ischemia, brain tumors, and hydrocephalus. Patients received anodal HD-tDCS or Sham stimulation using a Starstim8 stimulator (Neuroelectrics Corporation). The Starstim8 stimulator is a wireless and wearable eight-channel transcranial current stimulator with electroencephalogram (EEG) monitoring. Active electrode placement depends on which area of the brain is being stimulated. In this experiment, the cathode electrode was located in the right prefrontal region (FP2) for each group. Figure 2 demonstrates the electrode montage of two groups using a 10–10 electrode positioning system. For “Group_1,” the anodal target zone was on the left DLPFC with electrodes on F3, F1, FC3, and FC1 locations. For “Group_2,” the anodal target zone was on the dominant anterior temporal lobe (DATL) with electrodes on F7, FT9, FT7, and T9 locations. The prefrontal cortex is the main brain structure related to executive functions and includes the DLPFC, medial, and orbitofrontal/VMPFC regions. Although it is highly interconnected, it has been suggested that the DLPFC, specifically, is more specialized in working memory, a type of executive function [41]. The left anterior temporal lobe is referred to as the “semantic hub” since it is involved in storing and retrieving conceptual knowledge [42]. To create Fig. 2, the Neuroelectrics Instrument Controller engine (NIC2, v2.0.11.7) was used. NIC2 is a software that provides a secure way to define the stimulation parameters and monitor noninvasive brain stimulation. In the Stim Preview window, the electric field generated in the brain as a result of the stimulation montage is displayed in a 3D standardized model. It is often suggested to use the Stim Preview visualization as a confirmation step before applying the stimulation protocol. Stim Preview confirms that the chosen montage will target the desired brain area. It should be pointed out that there is no subject-level modeling to validate individual differences in the estimated e-field shown in Fig. 2.

**Fig. 3:**
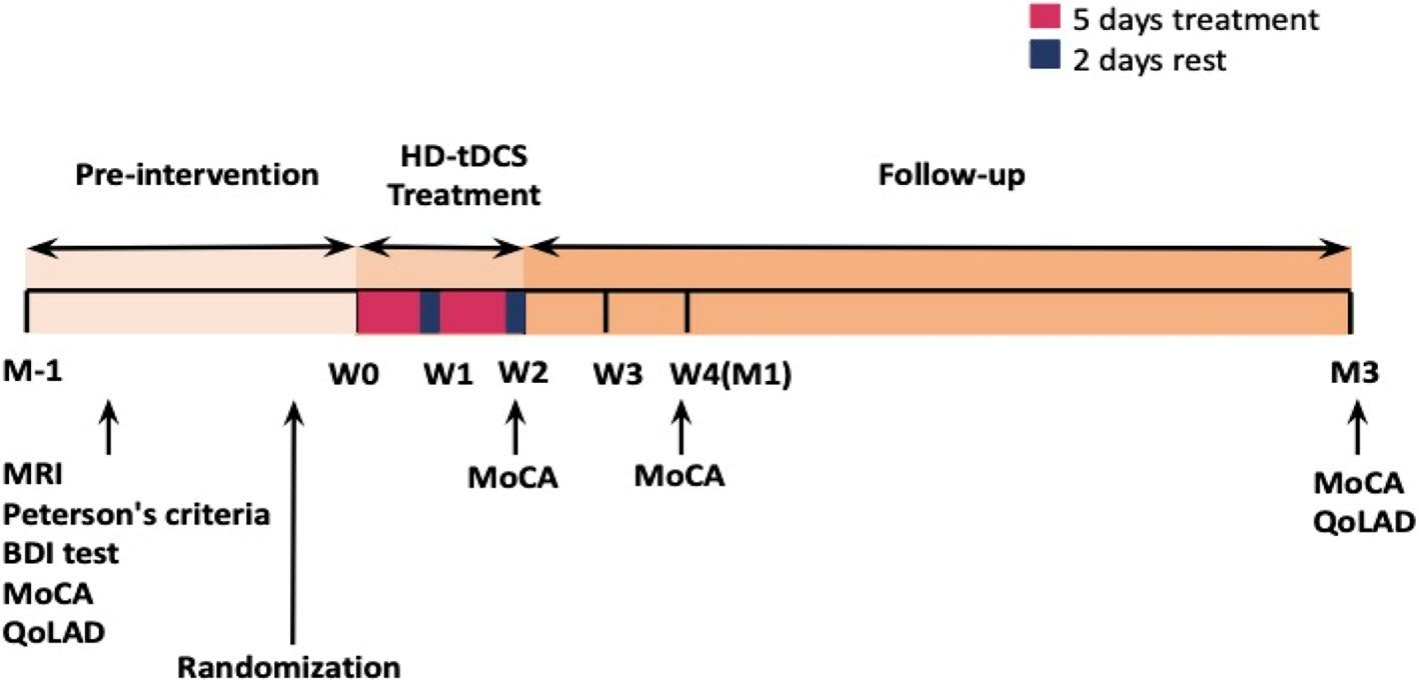
The study timeline explains three stages of assessments including pre-intervention (baseline), intervention (treatment), and follow-up (post-intervention). Pre-intervention period for 1 month, 2-week anodal HD-tDCS treatment period (W1, W2), and a 3-month follow-up period from the first day of stimulation. One month before treatment, MRI, Peterson’s criteria, and DBI tests were also done. Montreal Cognitive Assessment (MoCA) test was taken for each patient 1 month before treatment (M_-1_), the second week (W2), and the first (M1) and the third month (M3). Quality of Life in Alzheimer’s Disease (QoLAD) at baseline and the third month was measured

The timeline of this study is illustrated in Fig. 3. Both intervention groups received 20 min of HD-tDCS anodic stimulation of 2 mA on five successive days in a week for two consecutive weeks. In order to measure only stimulation effects in the MCI patients, we did not ask them to do anything during the stimulation time interval. Stimulation was delivered through five electrodes (PISTIM, circular Ag/AgCl electrode with a radius of 1 cm, and a contact surface of 3.14 cm^2^). The space between each electrode and scalp was filled with gel using a syringe. Special caps helped to fix the electrodes secured in place. The electrodes of this device were in the form of a low-diameter ring that allowed for increased penetration of electrical stimulation and more precise localization, thereby maximizing intensity and neuromodulation in the region to achieve the desired target and minimizing relative intensity outside of the targeted area.

**Fig. 4:**
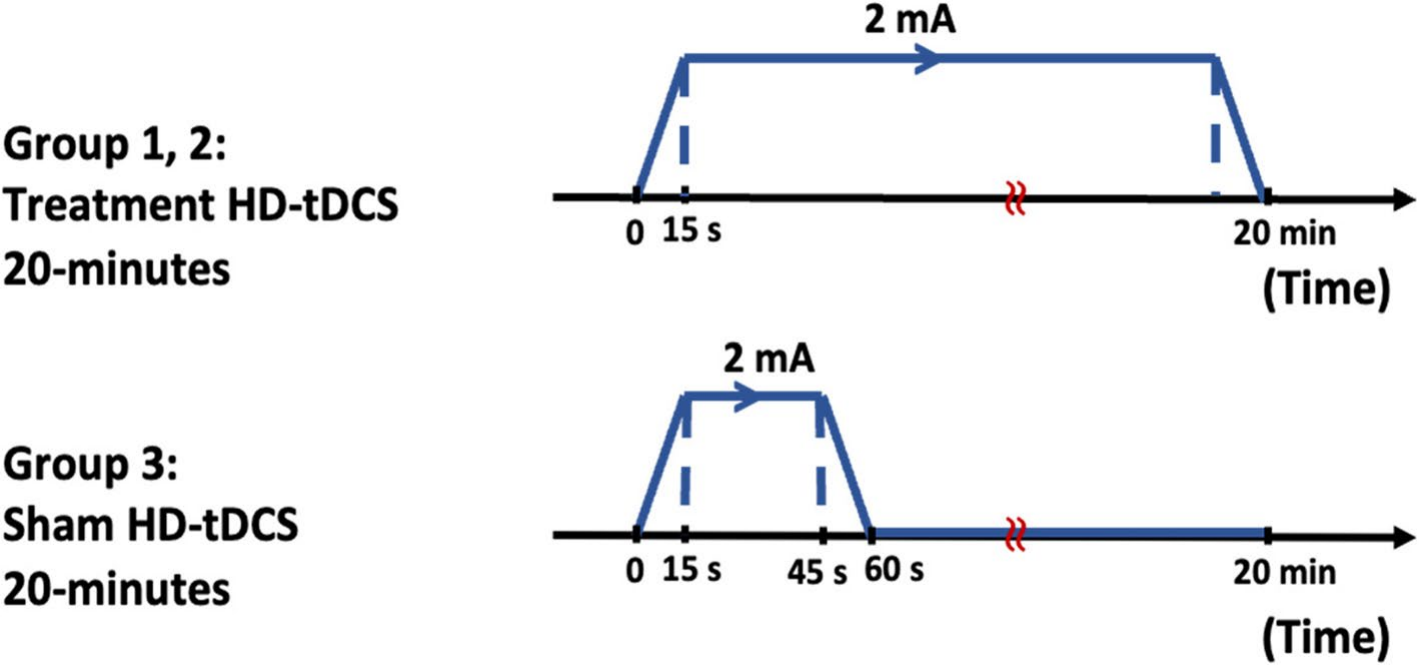
The experimental protocol of stimulation for intervention and Sham groups. **A** Intervention group. Patients received a direct current for 20 min. **B** Sham group. Patients received direct current for only 60 s, while electrodes remained for 20 min. During the first 15 s, the current flow increased slowly, and during the last 15 s, the current flow decreased slowly

Conducting a Sham session implies that the subject undergoing the experiment should not be aware of whether a real tDCS protocol is being applied or a fake tDCS protocol. In 20 patients for Sham stimulation, electrodes were placed in the same active stimulation position on the scalp, and the patient received stimulation for only 60 s. However, the electrodes remained in place for 20 min. The flow increased slowly in the first 15 s and decreased slowly in the last 15 s. Patients had a feeling of pruritus and murmur but received no flow for the remaining 19 min [43]. The experimental protocol of stimulation for the intervention and Sham groups has been shown in Fig. 4.

### Tools

#### The Beck test

Beck Depression Inventory (BDI) test has consisted of statements including cognitive, affective, somatic, and vegetative symptoms of depression [44]. This test was used to rule out depression in all subjects because depression can be an important differential diagnosis for MCI patients [45, 46]. This test was performed before intervention for all patients as an exclusion criterion. The BDI test is a 21-question multiple-choice self-report inventory. For each answer, a value of 0 to 3 is assigned, and the total score is compared with a key to determine the severity of the depression. In this research, participants with test scores higher than 4 on the BDI test were excluded from the study.

#### The Montreal Cognitive Assessment (MoCA) test

To evaluate possible memory improvement using HD-tDCS, the MoCA test was considered. This test was done during the baseline, in the second week, the first and the third months after intervention as illustrated in Fig. 3. The MoCA test includes 8 parts with a maximum score of 30 (a score of 26 and higher indicates normal). In this study, those subjects with scores between 17 and 25 on the MoCA test were considered MCI patients.

#### Quality of Life in Alzheimer’s Disease (QoLAD)

The patient self-administered version of QoLAD was a 13-item questionnaire designed to provide both a patient and a caregiver report on the quality of life of patients who have been diagnosed with Alzheimer’s disease [47, 48]. The QoLAD scores were performed before and 3 months after the stimulation for all groups.

### Statistical analysis

The data were described using mean and standard deviation and frequency. The Kolmogorov–Smirnov was utilized to check the normal distribution of the quantitative data. The analysis of variance (ANOVA) and chi-square tests were used to compare the baseline variables among the three groups. Furthermore, the analysis of covariance (ANCOVA) was employed to compare the quality-of-life status. In addition, two-way repeated measures ANOVA (RMANOVA) and 3 × 4 (groups × time intervals) were recruited to compare the response to treatment between the two groups at different time intervals. The dependent variables were the QoLAD and MoCA scores during time intervals. All statistical procedures were performed using SPSS statistical software (version. 26). A *p*-value of less than 0.05 in two-sided tests was considered statistically significant.

## Results

### Demographic characteristics of subjects

In the present study, 22 women and 38 men participated. The age of subjects was 68.88 ± 9.88 (mean ± SD) years. Less than half of the applicants were cigarette smokers (*n* = 24, 40%). Hypertension and diabetic mellitus were reported by more than one-quarter of subjects (*n* = 19, 31.67%, and *n* = 21, 35%, respectively). Table 1 shows the detailed demographic information of participants in different groups.

**Table 1:** The comparison of baseline characteristics of participants in the left DLPFC, DATL, and Sham groups

Variables	Group 1: Left DLPFC (*N* = 20)	Group 2: DATL (*N* = 20)	Group 3: Sham (*N* = 20)	*p*-value
Age, mean (SD)	68.25 (10.26)	69.05 (9.91)	69.35 (9.94)	NS
Female sex, (%)	6 (30)	7 (35)	9 (45)	NS
Male sex, (%)	14 (70)	13 (65)	11 (55)	NS
Less than diploma, (%)	10 (50)	9 (45)	10 (50)	NS
Cigarette smoker, (%)	7 (35)	9 (45)	8 (40)	NS
Hypertension, (%)	6 (30)	6 (30)	7 (35)	NS
Diabetic mellitus, (%)	5 (25)	8 (40)	8 (40)	NS

Left *DLPFC* left dorsolateral prefrontal cortex, *DATL* dominant anterior temporal lobe, *N* number of patients, *NS* non-significant

### Comparison of MoCA mean scores between groups

**Fig. 5:**
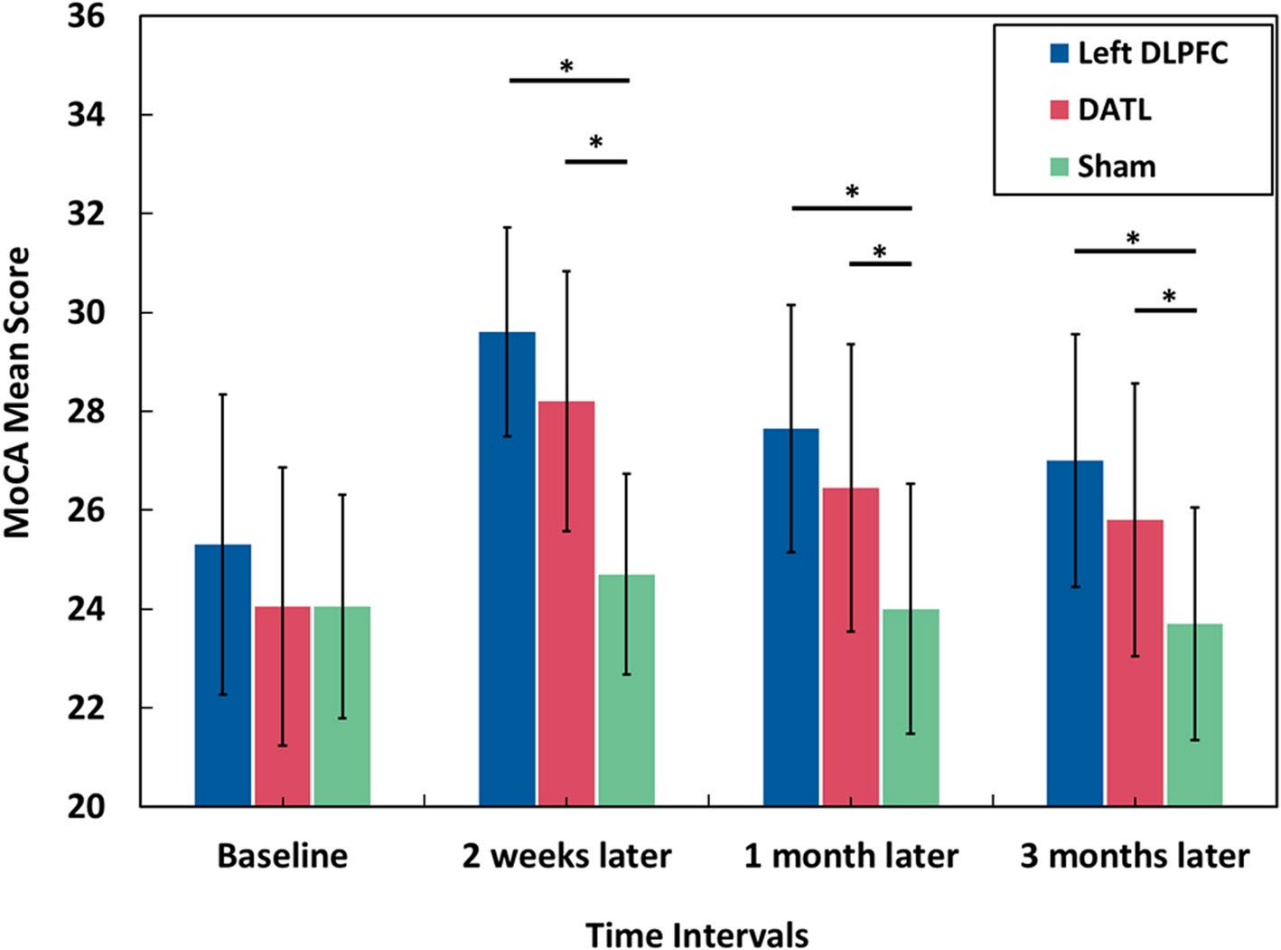
The MoCA mean scores between different time intervals: baseline, 2 weeks later, 1 month later, and 3 months later. Two weeks later, patients achieved highest scores in the MoCA test in the left DLPFC and DATL groups compared to the other time intervals. “*” shows the statistically significant cases (*p*-value ≤ 0.05). The data are presented using mean ± SD (standard deviation)

In the repeated measures analysis of variance (RMANOVA), the interaction effect is an important factor that needs to be examined. If there is a significant interaction between group and time, the marginal effects are investigated through post hoc tests. In this study, the interaction effect for MoCA mean score was significant (${F}_{(89.142, 217.475)}$ = 11.682, *p*-value = 0.001), and Bonferroni’s post hoc test was used to compare the difference between time intervals (Table 3 and 4). The effect size of the test was calculated through the partial eta square (${\eta }_{p}^{2}$). Tables 2 and 3 show that regarding the Sham group, the MoCA average values decreased from 24.05 at baseline to 23.70 after 3 months, which was not statistically significant. Table 3 and Fig. 5 illustrate that the MoCA mean scores have increased significantly (*p*-value ≤ 0.05) in both the left DLPFC and the DATL groups compared to the Sham group across all measurement intervals (2 weeks, 1 month later, 3 months later). However, for both the left DLPFC and the DATL groups, the MoCA mean scores have a decreasing trend from 2 weeks to 1 month and then to 3-month post-intervention (Table 2, Fig. 5). Regarding Table 3, it is also evident that between the left DLPFC and DATL groups, there was no statistically significant change in the MoCA mean scores in the different measurement intervals.

**Table 2:** Comparison of MoCA mean scores for each group in different time intervals

**Time intervals**	**Left DLPFC**	**DATL**	**Sham**	${{\varvec{\eta}}}_{{\varvec{p}}}^{2}$
**Baseline**	25.30 (3.04)	24.05 (2.82)	24.05 (2.26)	0.255
**2 weeks later**	29.60 (2.11)	28.20 (2.63)	24.70 (2.03)	
**1 month later**	27.65 (2.50)	26.45 (2.91)	24.00 (2.53)	
**3 months later**	27.00 (2.55)	25.80 (2.76)	23.70 (2.36)

**Table 3:** The Bonferroni post hoc tests of MoCA scores between groups for different times (*p-value ≤ 0.05)

Time intervals	Groups	DATL	Sham
**Baseline**	Left DLPFC	1.25	1.25
	DATL	-	0.01
**2 weeks later**	Left DLPFC	1.40	4.90*
	DATL	-	3.50*
**1 month later**	Left DLPFC	1.20	3.65*
	DATL	-	2.45*
**3 months later**	Left DLPFC	1.20	3.30*
	DATL	-	2.10*

### Comparison of MoCA mean scores in different time intervals

Considering Table 4 and Figure A1(supplementary information), in the left DLPFC group, the achieved MoCA scores in 2 weeks and 1 and 3 months after the intervention were statistically higher than the baseline score (*p*-value ≤ 0.05). Similarly, in the DATL group, patients significantly obtained higher MoCA mean scores in 2 weeks and 1 and 3 months after the intervention in comparison to the baseline of the study (*p*-value ≤ 0.05). There was no significant change in the achieved MoCA scores for patients in the Sham group during the same time intervals with respect to the study baseline.

**Table 4:** The Bonferroni post hoc tests of MoCA scores for different time intervals among three groups (*p-value ≤ 0.05)

Groups	Time intervals	2 weeks later	1 month later	3 months later
**Left DLPFC**	Baseline	− 4.30*	− 2.35*	− 1.70*
	2 weeks later	-	1.95*	2.60*
	1 month later	-	-	0.65
**DATL**	baseline	− 4.15*	− 2.40*	− 1.75*
	2 weeks later	-	1.75*	2.40*
	1 month later	-	-	0.65
**Sham**	baseline	− 0.65	0.05	0.35
	2 weeks later	-	0.70	1.00*
	1 month later	-	-	0.30

### Comparison of MoCA sub-scale scores

Using RMANOVA, we investigated the group effect (left DLPFC, DATL, and Sham), and we found that there was a significant effect on *visuospatial*, *naming*, *verbal*, and *abstract thinking* variables. In other words, the mean scores of three groups for the aforementioned variables are significantly different (*p*-value ≤ 0.05). The effect size of the RMANOVA test which has been calculated through the partial eta square (${\eta }_{p}^{2}$) showed that 22.2% (*visuospatial*), 10.5% (*naming*), 22% (*verbal*), and 11.9% (*abstract thinking*) of changes were influenced by the group effects (Table A1, supplementary information). The interaction effect of group and time was also significant for *attention* and *memory* variables (*p*-value ≤ 0.05). The marginal effects of time and group were reported in Tables A2 and A3 (supplementary information) to investigate the impact of interventions on these variables.

**Fig. 6:**
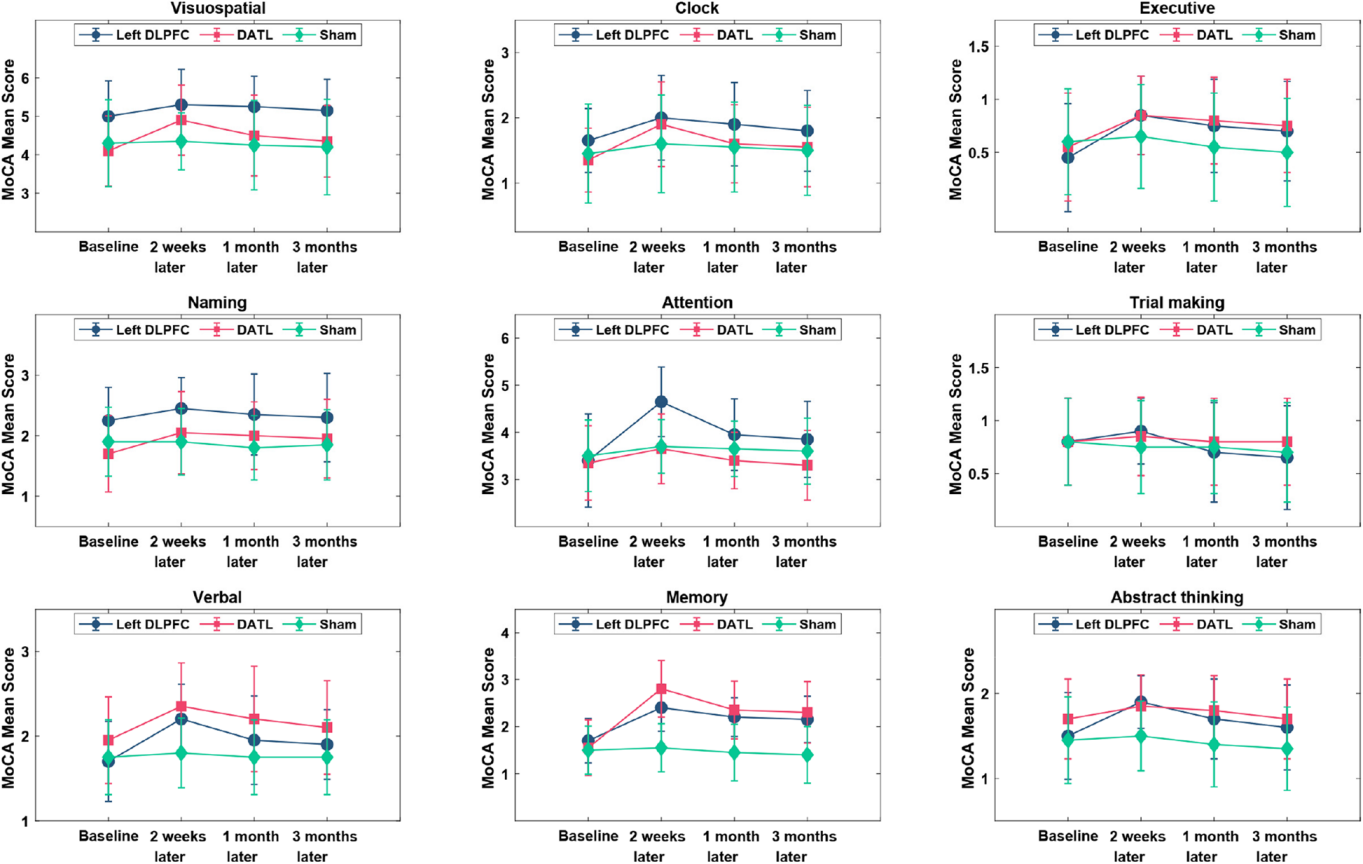
Comparison of MoCA mean sub-scale scores during different time intervals according to the groups. In general, the maximum score was observed after 2 weeks of intervention

Considering Table A2, for the left DLPFC group, patients obtained higher mean scores for both *attention* and *memory* components at 2 weeks, 1 month, and 3 months after the intervention compared to the baseline of the study (*p*-value ≤ 0.05). Moreover, in the DATL group, patients achieved higher mean scores for the *memory* component compared to baseline at 2 weeks, 1 month, and 3-month post-intervention (*p*-value ≤ 0.05). Considering Table A3, a significant difference was observed for the *attention* variable between the left DLPFC and DATL groups for 2 weeks and 1 and 3 months after the stimulation. However, between the left DLPFC with the Sham group only 2 weeks after the stimulation, this significant change was seen. Figure 6 shows, in the MoCA sub-scales, the maximum mean scores were observed 2 weeks after the intervention. There was no considerable change in the MoCA sub-scores in the Sham group.

### Comparison of QoLAD between groups

Table 5 demonstrates the results of the covariance analysis for QoLAD. We found that the main effects of the group (left DLPFC, DATL, and Sham) in QoLAD (${F}_{(2, 56)}$ = 22.355, *p*-value = 0.001, ${\eta }_{p}^{2}$ = 0.444) are significant. Indeed, in the left DLPFC and DATL groups, an increase in the QoLAD mean scores was observed after 3 months, highlighting the effectiveness of HD-tDCS in enhancing the quality of life in MCI patients. There was no considerable change in the QoLAD mean scores in the Sham group.

**Table 5:** Comparison of the covariance analysis test for QoLAD mean scores between groups

	**Baseline**	**3 months later**	**Group**
**Left DLPFC**	37.55 (3.24)	43.50 (3.02)	${F}_{(2, 56)}$= 22.355 *p* = 0.001 ${\eta }_{p}^{2}$ = 0.444
**DATL**	37.70 (3.20)	41.45 (3.61)	
**Sham**	37.40 (3.22)	37.85 (3.94)	

## Discussion

Since the initial investigation of tDCS in humans, many groups have attempted to further explore its applications. From a pathophysiological standpoint, tDCS works by delivering a low electrical current to the brain through scalp electrodes, altering neuronal membrane potentials. The underlying mechanisms involve modulation of synaptic plasticity and neuronal excitability, primarily through changes in the resting membrane potential. On the clinical side, several studies have also shown potential therapeutic applications of tDCS for various neurological and psychiatric diseases [14, 49, 50].

Among noninvasive brain stimulation techniques, there is more interest in using tDCS for cognitive decline in old age [32]. Previous studies have shown the DLPFC plays a role in executive function and control of cognitive tasks [51]. In this clinical research, we investigated the effect of HD-tDCS on the cognitive function of MCI patients by placing four anodal electrodes either over the left DLPFC or dominant anterior temporal lobe (DATL) and one cathode electrode over the supraorbital region. We have enrolled 60 MCI subjects that have been divided into 3 groups with 20 samples in each group. All 60 participants in 3 groups finished a 2-week intervention and a 3-month follow-up.

The results in the present study showed that after 2 weeks of intervention, the MCI patients in the left DLPFC and DATL groups achieved higher mean scores (from 25.30 to 29.60 and from 24.05 to 28.20, respectively) in the MoCA test versus the Sham group (from 24.05 to 24.70). Indeed, after 2 weeks, the left DLPFC group exhibited the most significant change (4.30), indicating a potentially substantial impact, while the DATL group showed a smaller change (4.15), suggesting a lesser influence. After 3 months, the MoCA mean score in the left DLPFC group was statistically higher than that of the Sham group (27.00 vs. 23.70, mean difference = 3.30). Therefore, the left DLPFC group is in a better condition compared to the Sham group in terms of the MoCA mean score.

Previous studies investigated the effect of tDCS on MCI patients. The results of a pilot study by Gonzalez and his colleagues showed that anodal tDCS over the left DLPFC leads to an improvement in cognitive performance of speed, selective attention, and working memory activities among MCI people [52]. In addition, Salehinejad and his collaborators confirmed that anodal tDCS over left DLPFC improved cognitive impairment [53]. According to our results, in both left DLPFC and DATL groups, the MoCA mean scores for 2 weeks and 1 and 3 months after the intervention were statistically higher than the baseline score (*p*-value < 0.05). Also, the MoCA score for 2 weeks after the intervention was higher than the other time intervals for both intervention groups (Fig. 5 and Figure A1). In parallel with the current research, Manor et al. showed a significant improvement in the MoCA score after 2 weeks through anodal tDCS intervention over the left DLPFC among older adults with functional limitations [54].

Regarding the comparison of MoCA sub-scale mean scores for different time intervals and according to the groups, the visuospatial, verbal, naming, and abstract thinking were statistically significant (*p*-value < 0.05). A significant interaction effect between group and time was observed for *attention* and *memory* variables (*p*-value < 0.05). Therefore, to investigate the effect of the interventions on these variables, the marginal effects of time and group were analyzed separately in Tables A2 and A3. In all three time intervals (2 weeks, 1 month, and 3 months), there was also a significant difference between the *memory* mean score of both the left DLPF and the DATL groups with the Sham group.

Boggio et al. showed that anodal tDCS on the left temporal cortex could improve visual recognition memory in Alzheimer’s disease [55]. Meanwhile, the results of another study by Boggio et al. demonstrated a significant improvement in visual cognitive memory after five consecutive sessions over a period of 5 days with anodal tDCS [29]. Andre et al. illustrated that using the anodal tDCS method over four consecutive sessions (for 20 min) on DLPFC had a positive effect on short-term visual memory, verbal memory, and control execution in patients with mild vascular dementia [35]. In addition, Manenti et al. showed a positive effect of tDCS on episodic memory among amnestic-MCI adults [56]. In parallel with these results, our findings highlight the potential of anodal HD-tDCS as one of the applicable noninvasive techniques that have positive effects on the cognitive function of MCI patients. Moreover, we compared the effects of brain stimulation in two different areas (left DLPFC, DATL) with Sham stimulation that may help detect the best target for tDCS stimulation in MCI patients. The cognitive function assessment was conducted not only after the intervention time but also to determine whether it had any significant effects in the long term; the follow-up was continued for 3 months.

## Limitations and future research

This study has some limitations that should be acknowledged. There is no individual modeling of the estimated e-fields to see how they matched up with the intended effects shown in Fig. 2. We suggest using MRIs of the patients to perform subject-level modeling of the estimated e-field or to look for structural changes resulting from the treatment. It should be noted that by placing the cathode at Fp2, the current could cause unwanted inhibition of some nearby areas; in contrast, some authors point out that extracranial cathodes increase the facilitation of some functions compared to the cranial cathodes as the supraorbital zone. It is a well-known principle of electromagnetism that current flow follows the path of least resistance. For intracranial current flow, this typically means the sutures and the orbit. In this way, the placement of the electrodes so close to the pterion and the orbit may have resulted in the stimulation outside of the intended region.

Although both patients and researchers (and the evaluating physician) were blinded, the technician who applied the tDCS therapy and the person who performed the statistical analysis were not blinded. We did not assess the blindness of participants and administrators which can be considered as another limitation of this study. Moreover, the original clinical trial was posted with 30 participants per group. However, in practice due to a lot of limitations, including COVID-19, and the number of MCI patients who agreed to attend the study, 20 participants per group were done.

Finally, another limitation is the transient effect of tDCS, which should be explored from the pathophysiological and clinical point of view in future works. This transient effect is thought to be related to alterations in the balance of inhibitory and excitatory neurotransmission, which can result in enhanced or reduced neuronal firing. However, the exact mechanisms and the duration of these effects are still subjects of ongoing research [57]. When considering clinical applications, it is crucial to understand the transient nature of tDCS-induced changes. Repeated sessions or maintenance protocols may be required to sustain the desired clinical outcomes over time. These points open new horizons for further research in brain stimulation and clarify for clinicians the limited effects, future preventions, and augmenting strategies regarding the transient effect of tDCS. Nevertheless, our study brings additional confirmation for the therapeutic potential of HD-tDCS on MCI patients. Larger studies are necessary to identify the optimal stimulation protocols and target regions.

## Conclusion

The present study investigated the effects of brain electrical stimulation in two different regions including left DLPFC (group 1) and DATL (group 2) intending to detect the best target for HD-tDCS in MCI patients. Our results suggested that HD-tDCS, as a non-pharmacological intervention, had a positive effect on the improvement of cognitive functions among MCI patients, although the duration of these effects was limited. This result facilitates the future applications of tDCS for both fundamental neuroscience and clinical research. It should be emphasized that the stimulation protocol including duration of stimulation time, electrode placement, and the number of stimulation sessions should be standardized to improve its applicability, and thus, further research is still necessary on the HD-tDCS. Finally, having individual models of current density over the real MRI of the participants and the EEGs before, during, and after stimulation provides better insight into the mechanism of the HD-tDCS. This is an interesting point that should be investigated in future studies.

### Supplementary Information

**Additional file 1: Figure A1.** In both left DLPFC and DATL groups, the achieved MoCA scores in two weeks, one, and three months after the intervention were statistically higher than the baseline score (*p*-value≤0.05). There was no significant difference in the achieved MoCA scores for patients in the Sham group during the same time intervals with respect to the study baseline. “*” shows the statistically significant cases regarding the baseline. The data are presented using mean ± SD (Standard Deviation). (MoCA: Montreal Cognitive Assessment, Left DLPFC: Left Dorsolateral prefrontal cortex, DATL: Dominant anterior temporal lobe). **Table A1.** Comparison of MoCA sub-scale mean scores between groups. **Table A2.** The Bonferroni post hoc tests of MoCA sub-scale mean scores during different time intervals between groups. **Table A3.** The results of Bonferroni post hoc test of MOCA sub-scale mean scores in different groups.

## Data Availability

All analyses reported in this work will be available from the corresponding author upon reasonable request.
