# Supplementary material for: Anodal HD-tDCS on the dominant anterior temporal lobe and dorsolateral prefrontal cortex: clinical results in patients with mild cognitive impairment
Source: Alzheimers Res Ther. 2024 Feb 3;16:27. doi: 10.1186/s13195-023-01370-y (PMC10837991; doi:10.1186/s13195-023-01370-y)
Supplement: Supplementary file 1 — Additional file 1: Figure A1. In both left DLPFC and DATL groups, the achieved MoCA scores in two weeks, one, and three months after the intervention were statistically higher than the baseline score (p-value≤0.05). There was no significant difference in the achieved MoCA scores for patients in the Sham group during the same time intervals with respect to the study baseline. “*” shows the statistically significant cases regarding the baseline. The data are presented using mean ± SD (Standard Deviation). (MoCA: Montreal Cognitive Assessment, Left DLPFC: Left Dorsolateral prefrontal cortex, DATL: Dominant anterior temporal lobe). Table A1. Comparison of MoCA sub-scale mean scores between groups. Table A2. The Bonferroni post hoc tests of MoCA sub-scale mean scores during different time intervals between groups. Table A3. The results of Bonferroni post hoc test of MOCA sub-scale mean scores in different groups. [file 13195_2023_1370_MOESM1_ESM.docx]

**Supplementary Information**

According to Figure A1, the MoCA mean scores were significantly changed with respect to the baseline for the left DLPFC and DATL groups during different time intervals (p-value≤0.05).


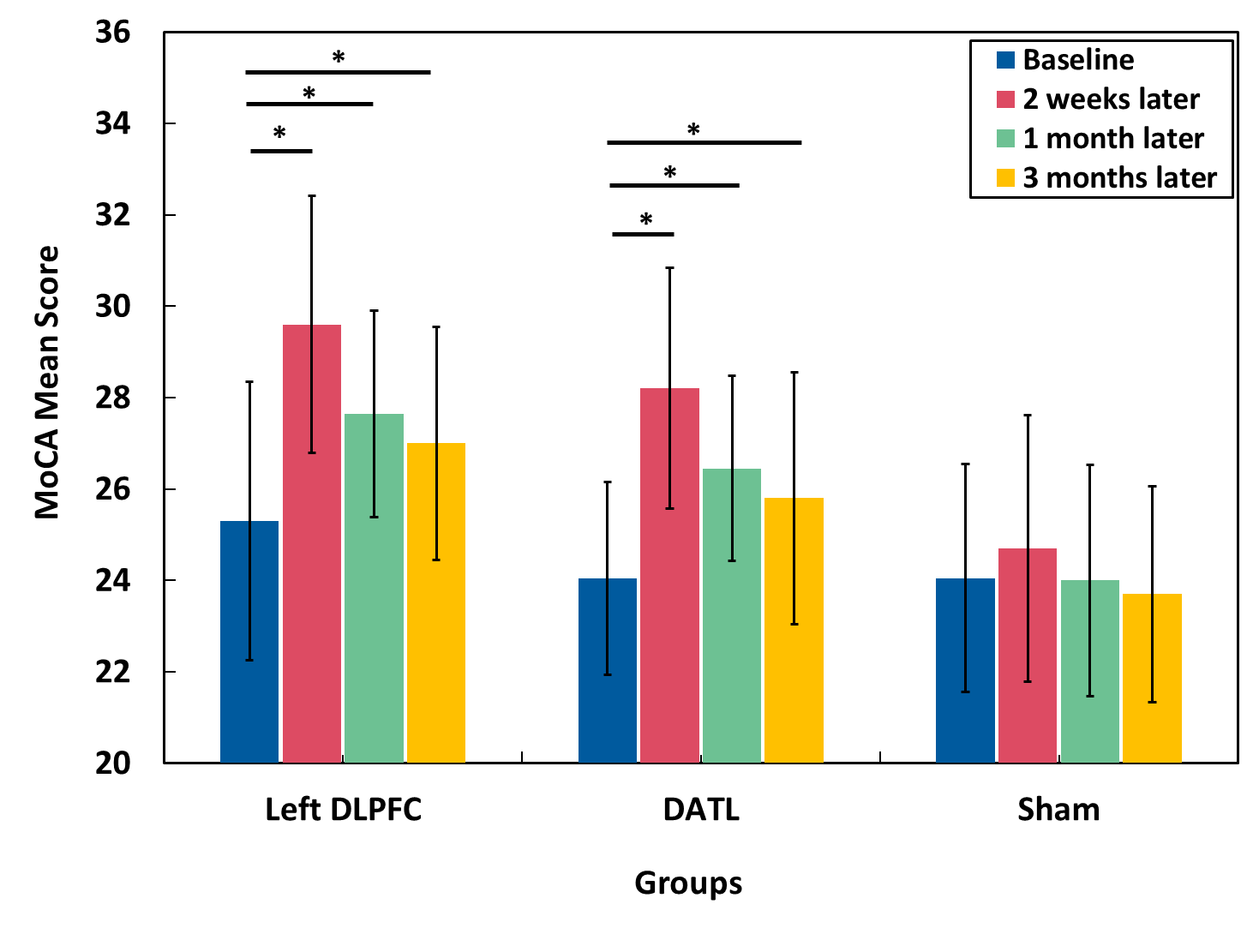


Figure A1. In both left DLPFC and DATL groups, the achieved MoCA scores in two weeks, one, and three months after the intervention were statistically higher than the baseline score (p-value≤0.05). There was no significant difference in the achieved MoCA scores for patients in the Sham group during the same time intervals with respect to the study baseline. “*” shows the statistically significant cases regarding the baseline. The data are presented using mean ± SD (Standard Deviation). (MoCA: Montreal Cognitive Assessment, Left DLPFC: Left Dorsolateral prefrontal cortex, DATL: Dominant anterior temporal lobe)

Table A1 shows the comparison of MoCA sub-scale mean scores during different time intervals according to the groups. The interaction effect of group and time is significant in *attention* and *memory* variables (p-value≤0.05). Consequently, the marginal effects of time and group to investigate the impact of interventions on these two variables are represented in Tables A2 and A3.

Table A1. Comparison of MoCA sub-scale mean scores between groups.

|  | **Time intervals** | **Left DLPFC** | **DATL** | **Sham** | **Interaction** | **Time** | **Group** |
| --- | --- | --- | --- | --- | --- | --- | --- |
| **Visu spatial** | Baseline | 5.00(0.92) | 4.10(0.91) | 4.30 (1.13) | F_(4,112)_= 1.539  P= 0.196 | F_(2,112)_= 8.428  P= 0.001  ^2^= 0.131η | F_(2,56)_= 8.011  P= 0.001  ^2^= 0.222η |
|  | 2 weeks later | 5.30 (0.92) | 4.90 (0.91) | 4.35 (0.74) |  |  |  |
|  | 1 month later | 5.25 (0.79) | 4.50 (1.05) | 4.25 (1.16) |  |  |  |
|  | 3 months later | 5.15 (0.81) | 4.35 (0.93) | 4.20 (1.24) |  |  |  |
| **Clock** | Baseline | 1.65 (0.49) | 1.35 (0.49) | 1.45 (0.76) | F_(4.798,136.738)_= 2.067  P= 0.076 | F_(2.399,136.738)_= 18.01  P=0.001  ^2^= 0.240η | F_(2,57)_= 1.531  P= 0.225  ^2^= 0.051η |
|  | 2 weeks later | 2.00 (0.65) | 1.90 (0.65) | 1.60 (0.75) |  |  |  |
|  | 1 month later | 1.90 (0.64) | 1.60 (0.60) | 1.55 (0.69) |  |  |  |
|  | 3 months later | 1.80 (0.62) | 1.55 (0.61) | 1.50 (0.69) |  |  |  |
| **Cube** | Baseline | 0.85 (0.37) | 1.00 (0.32) | 0.80 (0.41) | F_(4.534,129.206)_= 0.244  P=0.930 | F_(2.267,129.206)_= 0.974  P= 0.389  ^2^= 0.017η | F_(2,57)_= 2.333  P= 0.106  ^2^= 0.076η |
|  | 2 weeks later | 0.95 (0.22) | 1.00 (0.00) | 0.90 (0.31) |  |  |  |
|  | 1 month later | 0.90 (0.31) | 1.00 (0.00) | 0.85 (0.37) |  |  |  |
|  | 3 months later | 0.90 (0.31) | 1.00 (0.00) | 0.85 (0.37) |  |  |  |
| **Naming** | Baseline | 2.25 (0.55) | 1.70 (0.63) | 1.90 (0.57) | F_(4,112)_= 0.394  P= 0.813 | F_(2,112)_= 0.370  P= 0.691  ^2^= 0.007η | F_(2,56)_= 3.282  P= 0.045  ^2^= 0.105η |
|  | 2 weeks later | 2.45 (0.51) | 2.05 (0.68) | 1.90 (0.55) |  |  |  |
|  | 1 month later | 2.35 (0.67) | 2.00 (0.56) | 1.80 (0.53) |  |  |  |
|  | 3 months later | 2.30 (0.73) | 1.95 (0.65) | 1.85 (0.58) |  |  |  |
| **Executive** | Baseline | 0.45 (0.51) | 0.55 (0.51) | 0.60 (0.50) | F_(4.024,114.693)_= 2.014  P= 0.097 | F_(2.012,114.693)_= 7.486  P= 0.001  ^2^= 0.116η | F_(2,57)_= 0.923  P= 0.403  ^2^= 0.031η |
|  | 2 weeks later | 0.85 (0.37) | 0.85 (0.37) | 0.65 (0.49) |  |  |  |
|  | 1 month later | 0.75 (0.44) | 0.80 (0.41) | 0.55 (0.51) |  |  |  |
|  | 3 months later | 0.70 (0.47) | 0.75 (0.44) | 0.50 (0.51) |  |  |  |
| **Attention** | Baseline | 3.40 (0.99) | 3.35 (0.79) | 3.50 (0.76) | F_(4.838,137.884)_= 6.020  P=0.001 | - | - |
|  | 2 weeks later | 4.65 (0.74) | 3.65 (0.74) | 3.70 (0.57) |  |  |  |
|  | 1 month later | 3.95 (0.76) | 3.40 (0.60) | 3.65 (0.59) |  |  |  |
|  | 3 months later | 3.85 (0.81) | 3.30 (0.74) | 3.60 (0.70) |  |  |  |
| **Trial making** | Baseline | 0.80 (0.41) | 0.80 (0.41) | 0.80 (0.41) | F_(4.976,141.817)_= 0.850  P= 0.516 | F_(2.488,141.817)_= 2.099  P= 0.114  ^2^= 0.036η | F_(2,57)_= 0.180  P= 0.836  ^2^= 0.006η |
|  | 2 weeks later | 0.90 (0.31) | 0.85 (0.37) | 0.75 (0.44) |  |  |  |
|  | 1 month later | 0.70 (0.47) | 0.80 (0.41) | 0.75 (0.44) |  |  |  |
|  | 3 months later | 0.65 (0.49) | 0.80 (0.41) | 0.70 (0.47) |  |  |  |
| **Verbal** | Baseline | 1.70 (0.47) | 1.95 (0.51) | 1.75 (0.44) | F_(4.463,127.182)_= 1.208  P= 0.310 | F_(2.231,127.182)_= 6.373  P= 0.002  ^2^= 0.101η | F_(2,57)_= 8.033  P= 0.001  ^2^= 0.220η |
|  | 2 weeks later | 2.20 (0.41) | 2.35 (0.51) | 1.80 (0.41) |  |  |  |
|  | 1 month later | 1.95 (0.52) | 2.20 (0.62) | 1.75 (0.44) |  |  |  |
|  | 3 months later | 1.90 (0.41) | 2.10 (0.55) | 1.75 (0.44) |  |  |  |
| **memory** | Baseline | 1.70 (0.47) | 1.55 (0.59) | 1.50 (0.51) | F_(3.939,112.263)_= 7.794  P= 0.001 | - | - |
|  | 2 weeks later | 2.40 (0.50) | 2.80 (0.60) | 1.55 (0.51) |  |  |  |
|  | 1 month later | 2.20 (0.41) | 2.35 (0.61) | 1.45 (0.60) |  |  |  |
|  | 3 months later | 2.15 (0.49) | 2.30 (0.65) | 1.40 (0.60) |  |  |  |
| **Abstract thinking** | Baseline | 1.50 (0.51) | 1.70 (0.47) | 1.45 (0.51) | F_(6,171)_= 1.600  P= 0.155 | F_(3,171)_= 7.118  P= 0.001  ^2^= 0.111η | F_(2,57)_= 3.850  P= 0.027  ^2^= 0.119η |
|  | 2 weeks later | 1.90 (0.31) | 1.85 (0.37) | 1.50 (0.41) |  |  |  |
|  | 1 month later | 1.70 (0.47) | 1.80 (0.41) | 1.40 (0.50) |  |  |  |
|  | 3 months later | 1.60 (0.50) | 1.70 (0.47) | 1.35 (0.49) |  |  |  |
| **Orientation** | Baseline | 6.00 (0.00) | 6.00 (0.00) | 6.00 (0.00) | - | - | - |
|  | 2 weeks later | 6.00 (0.00) | 6.00 (0.00) | 6.00 (0.00) |  |  |  |
|  | 1 month later | 6.00 (0.00) | 6.00 (0.00) | 6.00 (0.00) |  |  |  |
|  | 3 months later | 6.00 (0.00) | 6.00 (0.00) | 6.00 (0.00) |  |  |  |

Table A2. The Bonferroni post hoc tests of MoCA sub-scale mean scores during different time intervals between groups.

| sub-scale | Variable | Time | 2 weeks later | 1 month later | 3 months later |
| --- | --- | --- | --- | --- | --- |
| Attention | Left DLPFC | Baseline | -1.25^*^ | -0.55^*^ | -0.45^*^ |
|  |  | 2 weeks later | - | 0.70^*^ | 0.80^*^ |
|  |  | 1 month later | - | - | 0.10 |
|  | DATL | Baseline | -0.30 | -0.05 | 0.05 |
|  |  | 2 weeks later | - | 0.25 | 0.35 |
|  |  | 1 month later | - | - | 0.10 |
|  | sham | Baseline | -0.20 | -0.15 | -0.10 |
|  |  | 2 weeks later | - | 0.05 | 0.10 |
|  |  | 1 month later | - | - | 0.05 |
| Memory | Left DLPFC | Baseline | -0.70^*^ | -0.50^*^ | -0.45^*^ |
|  |  | 2 weeks later | - | 0.20 | 0.25 |
|  |  | 1 month later | - | - | 0.05 |
|  | DATL | Baseline | -1.25^*^ | -0.80^*^ | -0.75^*^ |
|  |  | 2 weeks later | - | 0.45^*^ | 0.50^*^ |
|  |  | 1 month later | - | - | 0.05 |
|  | sham | Baseline | -0.05 | 0.05 | 0.10 |
|  |  | 2 weeks later | - | 0.10 | 0.15 |
|  |  | 1 month later | - | - | 0.05 |

Table A3. The results of Bonferroni post hoc test of MOCA sub-scale mean scores in different groups.

| sub-scale | Time | Variable | DATL | Sham |
| --- | --- | --- | --- | --- |
| Attention | Baseline | Left DLPFC | 0.05 | -0.10 |
|  |  | DATL | - | -0.15 |
|  | 2 weeks later | Left DLPFC | 1.00^*^ | 0.95^*^ |
|  |  | DATL | - | -0.05 |
|  | 1 month later | Left DLPFC | 0.55^*^ | 0.30 |
|  |  | DATL | - | -0.25 |
|  | 3 months later | Left DLPFC | 0.55^*^ | 0.25 |
|  |  | DATL | - | -0.30 |
| Memory | Baseline | Left DLPFC | 0.15 | 0.20 |
|  |  | DATL | - | 0.05 |
|  | 2 weeks later | Left DLPFC | -0.40 | 0.85^*^ |
|  |  | DATL | - | 1.25^*^ |
|  | 1 month later | Left DLPFC | -0.15 | 0.75^*^ |
|  |  | DATL | - | 0.90^*^ |
|  | 3 months later | Left DLPFC | -0.15 | 0.75^*^ |
|  |  | DATL | - | 0.90^*^ |
